# Dataset on the Mediterranean soils from the coastal region of the Lattakia governorate, Syria

**DOI:** 10.1016/j.dib.2020.105254

**Published:** 2020-02-07

**Authors:** Samar Ghanem, Adel Rukia, Magboul M. Sulieman, Eric C. Brevik, Safwan Mohammed

**Affiliations:** a Department of Soil and Water Science, Faculty of Agriculture, Tishreen University, Lattakia, Syria; b Department of Soil and Environment Sciences, Faculty of Agriculture, University of Khartoum, Khartoum North, 13314 Shambat, Sudan; c Soil Sciences Department, College of Food and Agricultural Sciences, King Saud University, P.O. Box 2460, Riyadh, 11451, Saudi Arabia; d Department of Natural Sciences, Dickinson State University, Dickinson, ND, USA; e Department of Agriculture and Technical Studies, Dickinson State University, Dickinson, ND, USA; f Institute of Land Use, Technology and Regional Development- Faculty of Agricultural and Food Sciences and Environmental Management-University of Debrecen, Debrecen 4032, Hungary

**Keywords:** Mediterranean soil, Soil classification, Mollisols, Vertisols, Land use planning, Syria

## Abstract

Soil survey is indispensable for land-use planning in any agro-ecosystem, particularly in coastal ecosystems because they often face several environmental problems such as flooding and water pollution, leading to soil degradation. The data given in this article revealing the common soil types and substantial taxonomy levels in the coastal region of Lattakia, Syria which is a key question for the land-use planning in the region. Data from 30 representative soil profiles and 60 auger points covering different agroecosystems within the Mediterranean coastal region of the Lattakia governorate, Syria were studied. The database including, the field morphological characteristics, physicochemical, mineralogical and micromorphological laboratory analyses. Entisols, Inceptisols, Mollisols, and Vertisols are the main soil types demonstrated in the area, which requiring convenient management for these divergent soils. The full profile data is available online in this data article for further reuse and for appropriate decisions to manage these soils.

**Table:** Specifications Table

Subject	Soil science
Specific subject area	Soil classification
Type of data	Table, image
How data were acquired	Soil morphological data were collected during a field soil survey conducted between 2017 and 2019 using the FAO guidelines for soil profile description. Laboratory data for physciochemical properties were acquired using standard soil methods. Rocks and soils mineralogical data were acquired using XRD and SEM instruments, while the soil chemical composition was determined using an X-ray fluorescence technique.
Data format	Raw, analysed
Parameters for data collection	Soil samples were air dried, ground (excluding rock fragments and concretions), screened through a 2 mm sieve and divided into representative subsamples using a riffle splitter. Rock samples were prepared in a 1.5x2 cm dimension for micromorphological analysis.
Description of data collection	A bout 106 soil and rock samples were collected from horizons of representative profiles. The soil samples were analysed for physicochemical properties using standard soil laboratory methods and selected soil and rock samples were analysed for mineralogical composition using XRD. Selected rock samples were analysed for micromorphological properties using a petrographic microscope.
Data source location	Lattakia Governorate, Syria (the rest coordinates for the studied sites are given in Table 1): LSYP01 35° 59′ 31.00"E 35° 38′ 48.00"N LSYP04 35° 55′ 40.00"E 35° 36′ 41.00"N LSYP10 35° 50′ 18.00"E 35° 33′ 56.00"N LSYP16 35° 52′ 32.00"E 35° 42′ 12.00"N LSYP21 35° 50′ 31.00"E 35° 36′ 34.00"N LSYP23 35° 48′ 38.21"E 35° 37′ 50.72"N LSYP26 35° 48′ 42.00"E 35° 35′ 47.00"N LSYP30 35° 50′ 20.88"E 35° 35′ 49.29"N
Data accessibility	The full dataset is given in this data article.

**Table:** 

**Value of the Data** • The data provide detailed information about soil development under the xeric and thermic soil moisture and temperature regimes, respectively, found in the Lattakia region, Syria. • Due to the absence of any soil database for Syria in the literature, this data provides a valuable source of soil information for the coastal region of Syria in particular. • This database of 30 soil profiles can be used by international database platforms such as ISRIC (International Soil Reference and Information Centre) and HWSD (Harmonized World Soil Database) to test the accuracy of their database at the regional scale. • As soil classification plays an important role in land use planning, this database could help decision makers and international organizations (FAO, ICARDA, ACSAD) design optimum land use plans for the rehabilitation stage after the Syrian War ends.

## 1 Data description

**Fig. 1:**
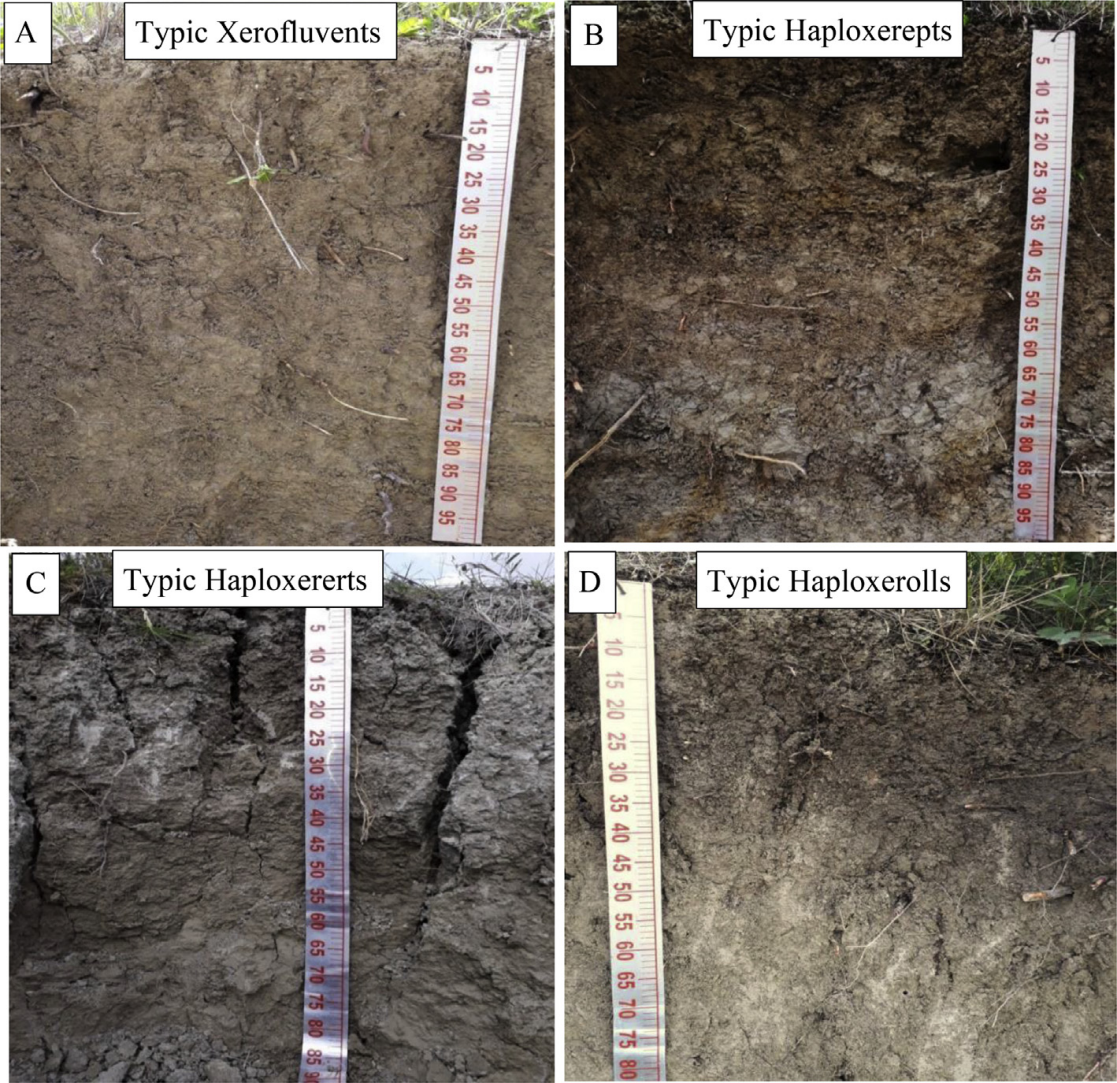
Selected representative soil profiles from the Lattakia region, Syria showing Entisols (A), Inceptisols (B), Vertisols (C), and Mollisols (D).

**Fig. 2:**
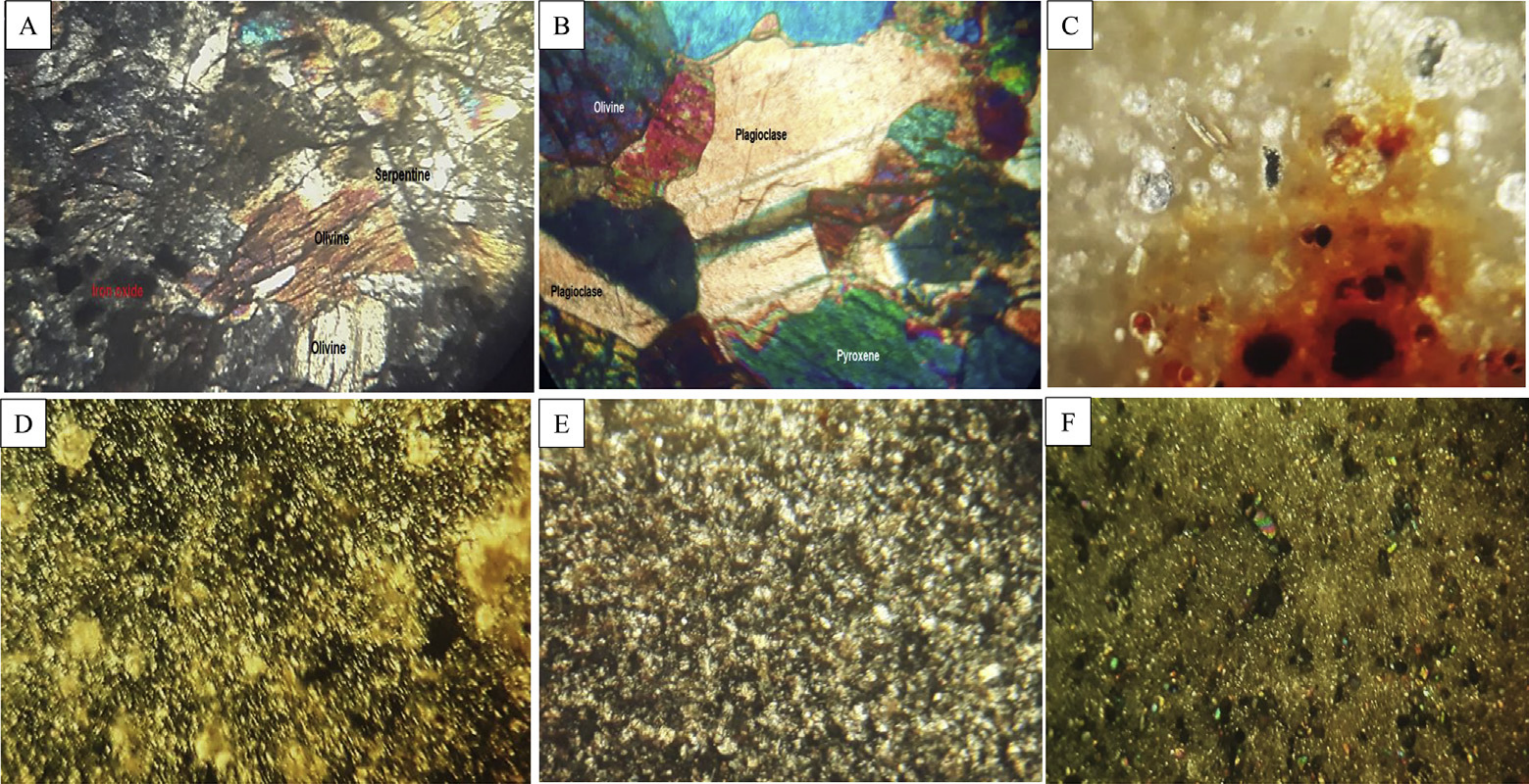
Selected rock thin sections under polarized light in a petrographic microscope showing minerals and other micromorphologically features: Olivine, serpentine, and Fe-oxides (A), olivine, pyroxene, and plagioclase (B), calcite with microfossils and Fe-oxides (C), fine calcite crystals (grayish color) with Fe-oxides (D), gypsum crystals (white color) and rare Fe-oxides (E), and fine sand particles (commonly plagioclase and pyroxene) associated with calcite crystals (F). Field of view in the horizontal direction is 40–50 μm with 40X magnification.

Fig. 1 shows some representative profiles associated with the main soil orders found in the coastal region of Lattakia governorate, Syria. Fig. 2 shows the micromorphology features for selected rock samples from the study area. Table 1 provides some key site properties for the selected representative soil profiles with their classification. Table 2 summarizes the field morphological descriptions for the selected soil profiles. Table 3 presents physiochemical soil properties for selected soil profiles. Table 4 gives the organic matter distribution and fractionations data for selected horizons from the soil profiles. Table 5 shows the minerals identified in the representative soil profiles based on XRD analysis. Table 6 provides data on the soil chemical composition of the representative soil profiles.

**Table 1:** Site description and soil classification for the selected representative profiles from the Lattakia governorate, Syria.

Profile	Coordinates (E/N)		Elevation (m)	Slope (%)	Parent material	Land cover	Land use	Soil classification**
LSYP01	35° 59′ 31.00"E	35° 38′ 48.00"N	165	20	Marlstone	Pine	Mixed Forest	Xerepts
LSYP02	35° 58′ 57.000"E	35° 37′ 27.000"N	133	30	Gypsum	Oak-Pine	Mixed Forest	Xerolls
LSYP03	35° 56′ 32.460"E	35° 37′ 12.495"N	51	15	Marlstone	Myrtus communis- Pine	Mixed Forest	Xerolls
LSYP04	35° 55′ 40.00"E	35° 36′ 41.00"N	51	10	Alluvial sediment deposit	Pine	Forest	Fluvents
LSYP05	35° 54′ 35.754"E	35° 36′ 22.111"N	35	5	Alluvial sediment deposit	Pine-Oak	Mixed Forest	Fluvents
LSYP06	35° 54′ 42.000″ E	35° 37′ 2.000″ N	81	35	Peridotite	Myrtus communis- Pine	Mixed Forest	Xerolls
LSYP07	35° 54′ 45.000″ E	35° 37′ 30.000"N	128	15	Gabbro	Myrtus communis- Oak	Mixed Forest	Orthents
LSYP08	35° 53′ 31.000″ E	35° 35′ 21.000"N	18	5	Marlstone	Citrus	Agricultural	Xererts
LSYP09	35° 51′ 15.000″ E	35° 32′ 55.000"N	50	30	Calcareous rock	Sumac-Cistus- Alhagi	Range land	Xerepts
LSYP10	35° 50′ 18.00"E	35° 33′ 56.00"N	90	40	Calcareous rock	Oak	Mixed Forest	Xerolls
LSYP11	35° 51′ 43.000″ E	35° 36′ 0.000″ N	140	10	Calcareous rock	Sarcopoterium- Cistus	Range land	Xerolls
LSYP12	35° 51′ 30.883″ E	35° 34′ 47.106"N	150	15	Marlstone	Myrtus communis- Oak- Alhagi	Mixed Forest	Orthents
LSYP13	35° 53′ 26.000″ E	35° 38′ 18.000"N	190	20	Calcareous rock	Pine-Oak- Cistus	Mixed Forest	Xerolls
LSYP14	35° 55′ 9.000″ E	35° 40′ 26.000"N	170	30	Marlstone	Pine-Oak- Cistus	Mixed Forest	Xerolls
LSYP15	35° 55′ 23.618″ E	35° 40′ 39.120"N	152	15	Mudstone	Pine-Sarcopoterium	Forest	Xererts
LSYP16	35° 52′ 32.00"E	35° 42′ 12.00"N	124	15	Marlstone	Pine	Mixed Forest	Orthents
LSYP17	35° 51′ 46.592″ E	35° 41′ 28.753"N	128	5	Marlstone	Citrus	Agricultural	Orthents
LSYP18	35° 50′ 12.444″ E	35° 41′ 28.753"N	109	20	Calcareous rock+ Quartz	Oak	Forest	Xerolls
LSYP19	35° 51′ 22.000″ E	35° 38′ 35.000"N	84	5	Alluvial sediment deposit	Citrus	Agricultural	Fluvents
LSYP20	35° 52′ 8.000″ E	35° 38′ 27.000"N	152	5	Calcareous rock	Olive	Agricultural	Orthents
LSYP21	35° 50′ 31.00"E	35° 36′ 34.00"N	102	10	Calcareous rock	Rock fragments	Range land	Orthents
LSYP22	35° 49′ 24.953″ E	35° 39′ 49.799"N	60	15	Alluvial sediment deposit	Citrus	Agricultural	Fluvents
LSYP23	35° 48′ 38.21"E	35° 37′ 50.72"N	75	30	Calcareous rock	Oak	Mixed Forest	Xerolls
LSYP24	35° 47′ 46.959″ E	35° 36′ 17.018"N	30	5	Alluvial sediment deposit	Citrus	Agricultural	Fluvents
LSYP25	35° 47′ 55.380″ E	35° 34′ 46.050"N	25	5	Alluvial sediment deposit	Citrus	Agricultural	Fluvents
LSYP26	35° 48′ 42.00"E	35° 35′ 47.00"N	30	30	Alluvial sediment deposit	Citrus	Agriculture	Fluvents
LSYP27	35° 49′ 18.174″ E	35° 34′ 34.410"N	100	30	Marlstone	Olive	Agriculture	Orthents
LSYP28	35° 51′ 26.404″ E	35° 37′ 19.662"N	190	10	Calcareous rock	Sarcopoterium- Cistus	Range land	Orthents
LSYP29	35° 50′ 45.629″ E	35° 36′ 48.824"N	120	20	Calcareous rock	Sarcopoterium- Cistus	Range land	Orthents
LSYP30	35° 50′ 20.88"E	35° 35′ 49.29"N	120	5	Marlstone	Olive	Agriculture	Xererts

** According to *Soil Taxonomy* (Soil Survey Staff, 2014).

**Table 2:** Morphological characteristics for the selected representative soil profiles from Lattakia, Syria.

Profile	Horizon	Depth (cm)	Matrix color	Texture Field^a^	Structure^b^	Roots^c^	HCl reaction^d^	Biological activity^e^	Horizon boundary^f^	Diagnostic horizon(s)
LSYP01	A	0–5	10YR 5/2	SL	1,F, GR	3	3	H	C, W	Ochric Cambic
	(B)	5–25	10YR 4/2	CL	2,M, B	2	3	M	G,SM	
	C	25–100	10YR 4/2	CL	3,M, B	1	3	WE	–	
LSYP02	A	0–20	7.5YR 5/3	SL	1,F, GR	3	2	H	G, W	Mollic
	AC	20–60	7.5YR 7/2	CL	1,F, GR	2	2	H	G, W	
	C	60–100	10YR 8/3	CL	2,F, B	0	2	M	–	
LSYP03	A	0–40	10Y 3/1	CL	1,F, GR	2	3	H	G, W	Mollic
	AC	40–75	7.5 Y5/2	CL	2,M, B	2	3	M	G, SM	
	C	75–120	10Y 4/1	CL	3,M, B	1	3	WE	–	
LSYP04	A	0–20	10 YR 4/1	SL	1,F, GR	3	3	H	D, W	Ochric
	C1	20–55	10 YR 4/2	SL	2,F, B	3	3	M	D, W	
	C2	55–100	10 YR 4/2	SL	3,M, B	1	3	WE	–	
LSYP05	A	0–45	10YR 2/1	CL	1,F, GR	3	4	H	C, W	Mollic
	C1	45–70	7.5YR 6/1	CL	1,F, GR	2	4	WE	C, W	
	C2	70–100	7.5YR 5/1	CL	2,M, B	1	4	WE	–	
LSYP06	A	0–20	2.5Y3/3	SL	1,F, GR	3	1	H	C, W	Mollic
	C	20–45	2.5Y3/3	SL	1,F, GR	2	1	M	C, W	
	R	45–100	–	–	–	–	–	–	–	
LSYP07	A	0–20	2.5Y3/3	SL	1,F, GR	2	1	H	A,W	Ochric
	AC	20–55	2.5Y5/2	L	1,F, GR	1	1	M	D,W	
	C	55–100	–	–	–	–	–	–	–	
LSYP08	Ap	0–10	10YR 4/1	CL	1,F, B	2	4	M	G,W	Anthropic
	AC	10–70	10YR 5/2	CL	2,M, ABK	1	3	WE	G,SM	
	C1	70–130	10YR 6/2	CL	3,M, ABK	1	2	WE	G,W	
	C2	130–160	10YR 6/2	CL	3,C, ABK	1	2	–	–	
LSYP09	A1	0–10	10YR 6/3	S	1,F, P	1	3	M	D,W	Ochric Cambic
	(B)	10–55	10YR 6/3	S	1,M, P	1	4	M	SM	
	BC	55–80	10YR 7/3	CL	2,M, P	1	4	M	C,W	
	C1	80–90	10YR 6/2	CL	2,M, B	1	4	WE	–	
	C2	90–110	10YR 7/2	CL	2,M, B	–	4	N	–	
LSYP10	A	0–20	10 YR 3/2	CL	1,F, GR	3	4	H	G,SM	Mollic
	AC	20–60	10YR 4/2	CL	1,M, GR	3	4	H	G,SM	
	C	60–100	10YR 6/2	CL	1,M, B	2	3	WE	–	
LSYP11	A	0–25	10YR 1.7/1	C	1,F, GR	3	4	H	C,W	Mollic
	R	25–70	10YR 4/2	–	–	–	4	–	–	
LSYP12	A	0–60	10YR 6/3	L	1,M, B	1	4	M	G,SM	Ochric
	AC	60–90	10YR 7/4	CL	2,M, B	1	4	WE	G,SM	
	C	90–130	10YR 7/4	C	2,M, B	0	4	–	–	
LSYP13	A	0–25	10YR 4/2	SCL	1,F, GR	3	3	H	G, W	Mollic
	AC	25–70	10YR 5/1	C	1,M, B	3	3	M	G,W	
	C	70–100	10YR 5/1	–	–	1	3	WE	–	
LSYP14	A	0–35	2.5Y3/1	L	1,F, GR	3	3	H	G,W	Mollic
	C	35–90	2.5Y5/3	C	2,M, B	2	3	–		
LSYP15	A	0–10	7.5Y 3/1	L	1,F, GR	3	1	H	G,W	Ochric
	C1ss	10–85	10 R 3/3	C	1,M, P	0	1	–	G,W	
	C2ss	85–100	10 R 3/3	C	3,C, P	0	0	–	–	
LSYP16	ˆA	0–30	10YR 5/2	CL	1,F, GR	3	3	H	G,W	NA
	A_b_	30–65	10YR 3/1	CL	1,F, GR	3	3	H	G, W	
	AC	65–100	10YR 5/2	CL	2,F, B	2	3	WE	–	
LSYP17	ˆA	0–25	2.5 Y 8/3	L	1,M, B	3	3	WE	A,W	NA
	ˆAC	25–85	2.5 Y 3/1	C	2,M, B	3	3	WE	G,IR	
	R	85–95	–	–	–	–	–	–	–	
LSYP18	A	0–17	10YR 1.7/1	CL	1,F, GR	3	2	H	G,W	Mollic
	AC	17–50	10YR 1.7/2	CL	1,M, GR	2	2	M	G,W	
	C	50–85	2.5Y 5/3	CL	2,M B	1	1	WE	G,W	
	R	85–110	–	–	–	–	–	–	–	
LSYP19	Ap	0–25	10YR 3/1	CL	1,F, GR	3	3	M	D,W	Ochric
	C	25–60	10YR 3/2	C	2,M, B	0	3	N	–	
LSYP20	A	0–10	10YR4/1	CL	2,M,MA	1	2	WE	G,W	Ochric
	CR	10–45	10YR5/1	C	3,M,MA	0	2	N	G,W	
	R	45–90	ROCK							
LSYP21	A	0–10	10YR 6/1	CL	1,F, GR	2	4	H	G,W	Ochric
	AR	10–30	10 YR 6/2	CL	2,M, B	1	4	WE	G,W	
	R	30–60		–	–	–	–	–	–	
LSYP22	A	0–20	5YR 3/4	C	1,F, GR	3	3	H	D,W	Ochric
	AR	20–50	5YR 4/2	C	2,M, B	3	3	M	D,SM	
	R	50–80	5YR 2/3	C	2,M, B	3	4	WE		
LSYP23	A_P_	0–25	10 R 2/1	SL	1,F, GR	3	3	H	G,W	Mollic
	AC	25–45	2.5YR 3/6	SL	1,F, GR	2	3	M	C,W	
	CR	45–65		–	–	0	3	0	G,W	
	R	65–85		–	–	0	3	0	–	
LSYP24	Ap	0–12	10YR 4/3	CL	1,F, GR	3	3	H	A, W	Ochric
	C1	12–22	5Y7/2	CL	2,F, GR	3	3	H	A, W	
	C2	22–100	10YR 3/2	C	2,M,B	2	3	M	–	
LSYP25	AP	0–20	10YR 1.7/1	CL	1,F, GR	1	3	WE	D,SM	Anthropic
	C	20–60	10YR 1.7/1	C	2,M,B	1	3	N	–	
LSYP26	Ap	0–20	10YR 2/1	CL	1,F, GR	3	2	H	C, W	Mollic
	C1	45–20	10YR 5/2	CL	2,F, GR	1	3	M	C, SM	
	C2	45–60	10YR 4/2	CL	2,M,SBK	1	3	WE	C, SM	
	C3	60–100	10YR 3/1	C	2,M,SBK	0	4	WE	–	
LSYP27	A	0–25	10YR6/2	CL	1,F, GR	3	3	M	G,W	Ochric
	AC	25–45	10YR6/2	CL	2,C, B	2	3	M	G,W	
	C	45–100	5Y6/1	C	ROCK	0	4	N	–	
LSYP28	A	30–0	10YR 6/1	CL	1,F, GR	3	3	M	G,W	Ochric
	AC	30–70	10YR 7/1	C	1,M, B	1	3	WE	G,W	
	R	100–70	ROCK							
LSYP29	A	0–17	2.5Y7/2	CL	1,F, GR	3	2	M	G,SM	Ochric
	CR	17–45	2.5Y 7/1	C	2,M,ABK	0	4	N	C,W	
	R	45–85	ROCK							
LSYP30	A	0–20	10YR 4/1	CL	3,M,ABK	1	3	WE	G, SM	Ochric
	C	20–85	10YR 4/1	CL	3,M,ABK	0	3	WE	–	

a Field texture: SL = sandy loam; CL = clay loam.

b Structure: 1 = weak; 2 = moderate; 3 = strong; F = fine; M = medium; C = coarse; SBK = subangular blocky; B = blocky, ABK = angular blocky; GR = granular; SG = Single grain; MA = massive.

c Roots abundance: 0 = none; 1 = few (2–20%); 2 = common (20–50%); 3 = many (>50%).

d HCl effervescence: 1 = slight; 2 = moderate; 3 = strong; 4 = very strong.

e Biological activity: N = none; W = weak; M = moderate.

f Horizon boundary: C = clear; D = diffuse; A = abrupt; SM = smooth; IR = irregular, W: wavy.

**Table 3:** Physiochemical soil characteristics of the selected representative soil profiles in the Lattakia region, Syria.

Profile	Horizon	Water content	BD	PD	clay	silt	sand	Texture	pH	EC	TOM	CaCO_3_	CEC	Ca^+2^	M^+2^	Na^+^	K^+^
		%	g/cm^3^		%			Class	H_2_O	d sm^-1^	%		Cmol_c_ kg^⁻1^				
LSYP01	A	6.32	0.90	2.39	31.70	63.80	4.50	SCL	7.80	0.16	8.44	74.61	54.10	44.00	8.20	0.90	0.50
	(B)	4.92	1.00	2.50	52.40	31.50	16.10	C	8.00	0.12	3.13	82.20	33.70	280.0	1.60	0.50	0.20
	C	5.04	1.13	2.50	47.10	36.69	16.20	C	8.00	0.12	0.64	70.63	37.50	33.80	1.40	1.50	0.20
LSYP02	A	10.5	0.91	2.36	14.26	71.88	13.88	SL	7.40	1.72	5.14	13.70	20.20	16.00	1.80	1.05	0.20
	AC	8.70	1.12	2.55	34.80	31.00	34.20	CL	7.40	1.66	2.52	25.80	19.00	12.60	4.60	1.83	0.30
	C	11.80	1.15	2.64	12.75	39.15	48.10	L	7.40	1.71	1.47	11.90	17.90	12.00	3.60	1.40	0.10
LSYP03	A	11.5	1.14	2.37	72.10	22.20	5.70	C	7.70	0.14	3.60	45.66	46.80	37.60	4.60	1.10	2.30
	AC	7.20	1.15	2.41	70.00	21.60	8.40	C	7.90	0.13	1.47	49.60	34.60	19.80	8.20	0.20	2.10
	C	2.70	1.23	2.49	69.30	20.50	10.2	C	8.00	0.13	0.27	56.36	48.60	21.80	25.2	0.10	0.70
LSYP04	A	4.38	–	2.52	23.12	36.53	40.35	L	8.00	0.09	4.10	47.35	28.00	22.80	2.00	0.50	1.90
	C1	5.08	–	2.63	25.50	29.32	45.18	L	8.10	0.09	1.88	46.44	21.00	14.00	4.20	0.30	1.60
	C2	5.50	–	2.76	34.64	15.01	50.35	SnC	8.30	0.08	3.34	40.61	23.00	16.60	3.10	0.10	1.40
LSYP05	A	5.70	1.41	2.51	15.00	8.20	76.80	SL	8.00	0.09	3.40	27.30	21.00	15.00	4.20	0.80	0.80
	C1	3.70	1.52	2.63	13.00	8.00	79.00	S	8.20	0.09	0.80	37.55	19.00	14.00	3.00	0.40	0.40
	C2	5.00	1.54	2.76	34.00	5.80	60.20	SnCL	8.10	0.08	1.00	38.89	25.00	19.40	4.00	0.60	0.60
LSYP06	A	5.70	1.02	2.10	41.86	15.85	42.29	C	7.90	0.10	7.40	13.70	56.10	36.00	17.2	0.80	1.30
	C	6.30	1.43	2.50	36.80	10.64	52.56	CSn	7.70	0.06	2.61	11.46	66.20	46.80	16.0	0.30	1.10
	R	–	–	–	–	–	–	–	–	–	–	13.93	–	–	–	–	–
LSYP07	A	5.08	1.27	2.66	49.80	19.98	30.22	C	7.60	0.12	4.01	15.95	38.00	29.40	5.20	1.40	1.30
	AC	3.18	1.58	2.71	17.95	4.13	77.92	SnC	7.70	0.04	3.66	17.36	32.60	20.20	8.60	1.20	1.20
	C	–	–	–	–	–	–	–	–	–	–	2.37	–	–	–	–	–
LSYP08	Ap	6.41	1.19	2.54	50.45	14.90	34.65	C	7.40	0.10	3.79	46.8	48.00	32.40	11.6	1.90	0.70
	AC	7.66	1.34	2.62	53.74	20.50	25.80	C	7.90	0.09	2.48	51.00	41.90	32.40	6.60	1.90	0.80
	C1	8.41	1.43	2.64	59.50	12.50	28.00	C	7.70	0.11	1.73	49.68	45.10	31.00	11.6	1.60	0.60
	C2	7.00	1.46	2.66	45.37	17.63	37.00	C	8.50	0.11	0.54	53.00	38.00	25.40	10.8	0.50	0.60
LSYP09	A	6.04	1.28	2.53	66.70	26.51	6.79	SC	7.80	0.09	3.19	50.99	40.40	30.00	5.70	1.10	2.10
	(B)	6.41	1.32	2.54	70.95	17.82	11.23	C	8.00	0.09	1.33	57.32	33.40	22.40	6.40	1.50	1.10
	BC	5.95	1.32	2.58	65.60	21.20	13.20	SC	8.00	0.12	1.04	53.92	22.60	13.00	6.00	2.10	1.30
	C1	5.30	1.35	2.58	65.19	22.64	12.17	SC	7.90	0.12	0.90	46.87	22.60	12.56	5.11	1.90	1.30
	C2	5.76	1.36	2.59	65.70	22.30	12.00	SC	8.00	0.09	0.50	60.07	26 .00	15.00	6.40	1.30	1.10
LSYP10	A	9.38	I.14	2.46	68.81	21.89	9.30	C	7.20	0.09	5.70	69.04	52.60	30.00	16.6	1.12	1.90
	AC	6.82	1.41	2.48	67.20	26.70	6.10	C	7.80	0.09	5.59	73.10	30.10	2500	2.40	1.11	1.18
	C	–	–	–	–	–	–	–	–	–	–	–	–	–	–	–	–
LSYP11	A	8.20	1.08	2.39	50.75	27.06	22.19	C	7.7	0.10	8.65	67.63	55.70	34.50	19.4	1.40	2.10
	R	–	–	–	–	–	–	–	–	–	–	95.29	–	–	–	–	–
LSYP12	A	6.32	1.38	2.36	66.38	26.39	7.23	SC	7.60	0.10	2.72	57.00	33.90	26.40	2.60	2.10	1.40
	AC	4.92	1.45	2.66	66.49	31.71	1.80	SC	7.90	0.12	1.82	57.42	37.10	17.80	10.3	1.00	0.60
	C	5.04	1.49	2.68	77.88	21.37	0.75	SC	8.00	0.17	1.68	49.92	42.50	21.20	12.8	1.20	0.70
LSYP13	A	7.39	1.43	2.46	65.95	20.85	13.20	C	8.20	0.12	2.90	48.16	33.40	24.60	3.00	0.70	2.40
	AC	9.68	1.58	2.49	67.36	21.94	10.70	C	8.00	0.12	2.15	51.54	35.00	29.60	0.80	0.90	2.40
	C	10.08	–	–	68.83	22.42	8.75	SC	8.30	0.13	0.72	59.08	41.60	22.80	7.60	1.40	1.60
LSYP14	A	7.30	1.43	2.65	71.86	27.05	1.09	SC	8.10	O.18	8.44	56.61	44.30	32.80	8.60	2.10	0.30
	C	9.60	1.52	2.68	70.07	25.93	4.00	SC	8.20	0.19	2.26	53.72	33.00	22.40	6.20	2.00	0.90
LSYP15	A	7.48	0.97	2.46	54.73	16.13	29.14	C	7.60	0.11	3.02	17.22	42.00	31.00	6.20	1.30	1.30
	C1ss	10.39	–	2.62	89.34	3.86	6.80	C	9.10	0.18	1.56	8.90	38.20	19.40	12.9	4.50	0.60
	C2ss	12.47	–	2.68	96.62	3.38	0.00	C	9.30	0.97	0.85	2.43	26.00	11.80	9.70	4.20	0.30
LSYP16	ˆA	5.62	1.36	2.47	53.00	26.95	20.05	C	8.10	0.09	3.50	56.98	41.20	31.00	7.00	0.90	0.60
	A_b_	5.79	1.20	2.21	70.26	10.58	19.16	C	7.80	0.07	5.37	52.42	44.10	33.00	7.80	1.10	0.70
	AC	4.32	1.45	2.62	70.42	11.46	18.12	C	8.10	0.06	2.50	59.25	35.20	25.00	6.00	1.00	0.30
LSYP17	ˆA	5.19	–	2.59	38.82	26.3	34.88	CL	8.10	0.10	2.90	66.05	24.60	15.00	2.30	2.40	1.90
	ˆAC	9.16	–	2.48	51.20	19.10	29.70	C	7.80	0.30	4.79	38.07	49.80	26.00	80.0	1.60	1.60
	R	–	–	–	–	–	–	–	–	–	–	78.08	–	–	–	–	–
LSYP18	A	11.32	0.92	2.43	76.67	2.82	20.52	C	7.10	0.06	9.00	15.86	87.00	67.40	14.2	2.40	1.10
	AC	12.76	–	2.60	69.28	3.98	26.74	C	7.70	0.06	4.82	37.58	83.10	64.60	13.0	2.30	1.30
	C	7.16	–	–	47.58	21.44	30.98	C	8.10	0.09	2.03	57.84	56.00	43.00	11.0	2.10	1.20
	R								–	–	–	72.02	–	–	–	–	–
LSYP19	Ap	8.19	1.28	2.53	61.58	10.82	27.6	C	8.30	0.70	4.32	51.20	38.00	26.90	7.40	0.30	2.30
	C	9.10	1.30	2.58	51.17	13.64	35.19	C	8.30	0.90	2.70	52.86	38.10	27.60	7.40	0.40	2.10
LSYP20	A	7.45	–	2.53	55.77	24.75	20.48	C	8.10	0.10	1.36	58.12	29.00	19.30	6.40	1.10	0.50
	CR	6.28	–	2.55	53.62	17.73	28.65	C	8.20	0.10	0.10	61.93	24.10	17.80	5.80	0.20	0.60
	R	–	–	–	47.43	22.72	29.58	C	–	–	–	60.04	–	–	–	–	–
LSYP21	A	6.53	1.23	2.41	65.42	26.64	7.94	S C	8.00	0.30	3.34	78.46	49.52	37.60	9.20	0.90	1.10
	AR	6.33	1.43	2.50	65.31	21.28	13.41	C	7.90	0.10	1.05	61.47	43.05	29.20	8.70	0.80	0.70
	R	–	–	–		–	–	–	–	–	–	–	–	–	–	–	–
LSYP22	A	6.53	1.23	2.41	65.42	26.64	7.94	SC	8.00	0.30	3.34	78.46	49.52	37.60	9.20	0.90	1.10
	AR	6.33	1.43	2.50	65.31	21.28	13.41	C	7.90	0.10	1.05	61.47	43.05	29.20	8.70	0.80	0.70
	R	–	–	–		–	–	–	–	–	–	85.00	–	–	–	–	–
LSYP23	Ap	10.52	1.55	2.65	30.84	38.70	30.46	CL	7.70	0.10	8.70	61.02	48.40	42.00	4.00	0.70	1.20
	AC	9.06	1.61	2.69	14.35	48.82	36.83	L	8.00	0.11	1.45	75.68	27.10	24.00	2.00	0.50	0.30
	CR	–	–	–	–	–	–	–	–	–	–	–	–	–	–	–	–
	R	–	–	–	–	–	–	–	–	–	–	–	–	–	–	–	–
LSYP24	Ap	10.23	1.04	2.40	55.50	38.90	5.60	C	8.00	0.14	9.58	71.09	42.70	34.40	2.80	2.20	1.60
	C1	11.53	1.19	2.51	53.30	14.10	32.60	C	8.50	0.10	2.61	84.34	38.30	30.40	3.20	1.70	1.60
	C2	11.40	1.22	2.53	56.00	16.90	27.10	C	8.10	0.16	1.30	31.66	49.80	43.60	4.80	0.20	0.60
LSYP25	AP	11.66	1.26	2.47	65.81	17.50	16.69	C	7.70	0.10	5.81	23.23	69.00	59.60	5.60	1.80	1.90
	C	11.23	1.23	2.59	58.21	11.32	30.47	C	8.00	0.11	1.45	24.05	67.10	56.80	5.60	1.60	1.50
LSYP26	Ap	9.05	1.63	2.59	66.95	16.36	16.69	C	8.00	0.10	1.53	44.09	48.50	36.70	8.12	0.20	1.60
	C1	7.93	–	2.63	55.47	21.59	22.94	C	8.10	0.10	0.72	70.70	37.90	30.00	4.00	0.20	1.20
	C2	8.50	–	2.64	62.90	11.10	26.00	C	8.00	0.10	0.87	56.16	39.00	33.00	3.00	1.50	1.40
	C3	9.53	–	2.67	71.20	20.10	8.70	C	8.00	0.10	1.45	63.13	40.20	34.00	4.00	0.40	1.40
LSYP27	A	10.12	–	2.66	63.21	20.93	15.86	C	7.90	0.10	5.11	61.00	43.00	30.00	6.40	2.60	1.80
	AC	11.06	–	2.76	59.27	27.15	13.58	C	8.00	0.11	1.68	76.54	36.90	24.40	7.80	2.90	1.60
	C	–	–	–	–	–	–	–	–	–	–	56.88	–	–	–	–	–
LSYP28	A	6.50	1.02	2.43	52.63	22.91	24.46	C	7.90	0.12	5.46	65.71	33.00	28.40	2.00	0.30	0.10
	AC	6.60	–	2.62	52.54	23.83	23.63	C	8.10	0.13	2.12	78.02	33.00	26.40	3.50	0.20	0.10
	R	–	–	–	–	–	–	–	–	–	–	–	–	–	–	–	–
LSYP29	A	7.10	1.78	2.65	19.00	17.50	63.20	C	8.00	0.01	1.24	64.03	27.50	23.60	2.80	0.80	0.80
	CR	4.06	–	–	22.70	19.60	38.20	CL	8.10	0.10	0.95	69.50	19.00	14.30	2.00	0.40	0.30
	R	–	–	–	–	–	–	–	–	–	–	72.13	–	–	–	–	–
LSYP30	A	8.85	–	2.73	69.56	15.25	15.19	C	7.70	0.50	1.54	66.77	42.60	33.20	4.60	1.60	2.00
	C	9.45	–	2.79	67.76	5.47	26.77	C	8.00	0.60	0.95	50.63	46.00	36.00	5.20	1.80	2.10

BD = Bulk density, PD = practical density, Sn = sand, C = clay, S = silty, L = loam, EC = Electrical conductivity, TOM = Total organic matter, CEC = Cation exchange capacity by 1M NH_4_OAc (pH = 7.0).

**Table 4:** Organic matter fractionation by horizon for the selected representative soil profiles in the Lattakia region, Syria.

Profile	Horizon	CH	CF	C	CH/CF	Hummus type	CH/C%*100	Humification level	CF+CH	Humin
		%								%
LSYP01	A	1.50	1.80	4.90	0.83	Fulvic- humic	30.61	High	67.35	32.65
	B	1.40	1.50	3.01	0.93	Fulvic- humic	46.51	Very high	96.35	3.65
LSYP02	A	1.30	1.10	3.02	1.18	humic -Fulvic	43.05	Very high	79.47	20.53
	AC	1.20	1.50	3.02	0.80	Fulvic- humic	39.74	High	89.4	10.60
LSYP03	A	0.70	0.90	2.11	0.78	Fulvic- humic	33.18	High	75.83	24.17
	C1	0.40	0.20	0.86	2.00	Humic	46.51	Very high	69.77	30.23
LSYP04	A	0.90	1.10	2.40	0.82	Fulvic- humic	37.50	High	83.33	16.67
	C1	0.60	0.30	1.10	2.00	Humic	54.55	Very high	81.82	18.18
LSYP05	A	0.40	0.30	2.00	1.33	Humic- Fulvic	20.00	Low	35.00	65.00
	C1	0.10	0.30	0.47	0.33	Fulvic	21.28	Average	85.11	14.89
LSYP06	A	1.00	1.20	4.30	0.83	Fulvic- humic	23.26	Average	51.16	48.84
	C	0.50	0.60	1.53	0.83	Fulvic- humic	32.68	High	71.90	28.10
LSYP07	A	1.20	0.70	2.30	1.71	Humic -Fulvic	52.17	Very high	82.61	17.39
	AC	0.80	0.50	2.15	1.60	Humic -Fulvic	37.21	High	60.47	39.53
LSYP08	A	1.20	0.80	2.20	1.50	Humic -Fulvic	54.55	Very high	90.91	9.090
	AC	0.20	0.10	1.45	2.00	Humic	13.79	Low	20.69	79.31
LSYP09	A	0.80	0.50	1.80	1.60	Humic -Fulvic	44.44	Very high	72.22	27.78
	(B)	0.50	0.20	0.78	2.50	Humic	64.10	Very high	89.74	10.26
LSYP10	A	1.30	0.80	3.30	1.63	Humic -Fulvic	39.39	High	63.64	36.36
	AC	1.40	0.60	3.20	2.33	Humic	43.75	High	62.50	37.50
LSYP11	A	1.20	0.90	5.00	1.33	Humic -Fulvic	24.00	Average	42.00	58.00
	R	–	–	–	–	–	–	–	–	–
LSYP12	A	0.70	0.60	1.60	1.17	Fulvic- humic	43.75	High	81.25	18.75
	C1	0.70	0.20	1.07	3.50	Humic	65.42	Very high	84.11	15.89
LSYP13	A	1.20	1.60	4.80	0.75	Fulvic- humic	25.00	Average	58.33	41.67
	AC	0.80	0.60	1.70	1.33	Humic -Fulvic	47.06	Very high	82.35	17.65
LSYP14	A	1.70	0.90	4.96	1.89	Humic -Fulvic	34.27	High	52.42	47.58
	C	0.60	0.20	1.32	3.00	Humic	45.45	Very high	60.61	39.39
LSYP15	A	0.60	0.90	1.70	0.67	Fulvic- humic	35.29	High	88.24	11.76
	C1SS	0.50	0.20	0.91	2.50	Humic	54.95	Very high	76.92	23.08
LSYP16	ˆA	1.00	0.50	2.05	2.00	Fulvic- humic	48.78	Very high	73.17	26.83
	A b	1.50	0.60	3.15	2.50	Humic	47.62	Very high	66.67	33.33
LSYP17	ˆA	0.70	0.80	1.70	0.88	Fulvic- humic	41.18	Very high	88.24	11.76
	CR	0.70	0.50	2.810	1.40	Humic -Fulvic	24.91	Average	42.70	57.30
LSYP18	A	1.80	1.30	5.20	1.38	Humic -Fulvic	34.62	High	59.62	40.38
	AC	1.70	0.70	2.83	2.43	Humic	60.07	Very high	84.81	15.19
LSYP19	A	1.30	0.80	2.50	1.63	Humic -Fulvic	52.00	Very high	84.00	16.00
	C	1.00	0.50	1.58	2.00	Humic	63.29	Very high	94.94	5.06
LSYP20	A	0.40	0.30	0.80	1.33	Fulvic- humic	50.00	Very high	87.50	12.50
	CR	0.20	0.30	0.68	0.67	Fulvic- humic	29.41	Average	73.53	26.47
LSYP21	A	0.80	0.70	1.90	1.14	Humic -Fulvic	42.11	Very high	78.95	21.05
	AR	0.30	0.20	0.60	1.50	Humic -Fulvic	50.00	Very high	83.33	16.67
LSYP22	A	0.90	0.80	2.50	1.13	Humic -Fulvic	36.00	High	68.00	32.00
	C1	0.70	0.50	1.91	1.40	Humic -Fulvic	36.65	High	62.83	37.17
LSYP23	A	0.30	0.10	5.11	3.00	Humic	5.87	Low	7.83	92.17
	C	0.40	0.20	0.85	2.00	Humic	47.06	Very high	70.59	29.41
LSYP24	A	2.10	0.80	5.60	2.63	Humic	37.50	High	51.79	48.21
	C1	0.60	0.80	1.53	0.75	Fulvic- humic	39.22	High	91.50	8.50
LSYP25	A	1.50	0.60	3.40	2.50	Humic	44.12	Very high	82.35	41.18
	C	0.40	0.30	0.85	1.33	Humic -Fulvic	47.06	Very high	94.12	5.88
LSYP26	A	0.20	0.10	0.30	2.00	Humic	66.67	Very high	100.00	0.00
	C1	0.20	0.10	0.42	2.00	Humic	47.62	Very high	71.43	28.57
LSYP27	A	1.20	1.10	3.00	1.09	Fulvic- humic	40.00	Very high	76.67	23.33
	AC	0.60	0.30	0.98	2.00	Humic	61.22	Very high	91.84	8.16
LSYP28	A	1.10	0.80	3.20	1.38	Humic -Fulvic	34.38	High	59.38	40.63
	AC	0.50	0.40	1.24	1.25	Humic -Fulvic	40.32	Very high	72.58	27.42
LSYP29	A	0.50	0.30	1.00	1.67	Humic -Fulvic	50.00	Very high	80.00	20.00
	C	0.50	0.20	0.72	2.50	Humic	69.44	Very high	97.22	2.78
LSYP30	A	0.40	0.20	0.90	2.00	Humic	44.44	Very high	66.67	33.33
	C	0.30	0.20	0.55	1.50	Humic -Fulvic	54.55	Very high	90.91	9.090

CH = carbon of humic acids, CF = carbon of fulvic acids.

**Table 5:** Minerals identified in the selected representative soil profiles by XRD analysis.

Profile	Horizon	Soil minerals
LSYP01	A	Calcite > Kaolinit>Enstatite>Illite>Saponite
	B	Calcite > Quartz > Gismondine > Enstatite > Pillipsite
	C	Calcite > Pillipsite > Leucite > Enstatite
LSYP02	A	Calcite > Quartz > Enstatite > Leucite
	AC	Calcite > Quartz > Enstatite > Leucite
	C	Calcite > Pillipsite > Leucite > Montmorillonite
LSYP03	A	Calcite > Quartz > Leucite > Saponite
	AC	Calcit > Quartz > Leucite > Enstatite > Saponite > Philipsite
	C	Calcite > Quartz > Diopside > Enstatite > Philipsite
LSYP04	A	Calcite > Kaolinite > Enstatite >. Leucite
	C1	Calcite > Quartz > Gismondine > Perlialite
	C2	Calcite > Quartz > Pillipsite > Perlialite
LSYP05	A	Calcite > quartz > Augite > Kaolinite
	C1	Calcite > Quartz > Gismondine > Montmorillonite.
	C2	Calcite > Quartz > Philipsitee > Perlialite
LSYP06	A	Leucite > Kaolinit > Philipsite.
	AC	Kaolinite > Philipsite > Leucite > Augite
	R	Quartz > philipsite > Diopside > Augite
LSYP07	A	Diopside > Philipsite > Augite > Enstatite
	AC	Enstatite > Philipsite > Kaolinite > Diopside > Augite.
	C	Anorthite > Diopsid > Philipsite
LSYP08	Ap	Calcite > Quartz > Philipsite > Gismondine
	AC	Calcite > Quartz > Leucite > Montmorillonite
	C1	Calcite > Quartz > Gismondine > Enstatite.
	C2	Calcite > Quartz > Leucite > Enstatite.
LSYP09	A1	Calcite > Quartz > Enstatite > Montmorillonite
	(B)	Calcite > Kaolinite > Philipsite > Enstatite.
	BC	Calcite > Enstatite > Leucite > Illite
	C1	Calcite > Philipsite > perlialite > Illite.
	C2	Calcite > Enstatite > Leucite > Illite
LSYP10	A	Calcite > Quartz > Gismondine > Montmorillonite
	AC	Calcite > Forsterite > Illite
	C	Calcite > Quartz > Leucite > Gismondine
LSYP11	A	Calcite > Enstatite > Illite > Montmorillonite
	R	Calcite > Quartz > Saponite > Montmorillonite
LSYP12	A	Calcite > leucite > Philipsite > Forsterite
	AC	Calcite > Quartz > Montmorillonite
	C	Calcite > Quartz > Kaolinite > Leucite
LSYP13	A	Calcite > Montmorillonite > Kaolinite > Illite
	AC	Calcite > Enstatite > Leucite > Anorthite-Clinocloss
	C	Calcite > Enstatite > Leucite > Kaolinite
LSYP14	A	Enstatite > Kaolinite- Leucite > Clinocloss
	C	Enstatite > leucite > Perlialite
LSYP15	A	Calcite > Quartz > Pillipsite > kaolinite
	C1ss	Quartz > kaolinite > leucite > Montmorillonite
	C2ss	Quartz > kaolinite > Pillipsite > Montmorillonite
LSYP16	A	Calcite > Quartz > Leusite > Philipsite
	Ap	Calcite > Quartz > Illite > Saponite
	AC	Calcite > Quartz > Pillipsite > Montmorillonite
LSYP17	A	Calcite > Quartz > Kaolinite > Montmorillonit
	AC	Calcite > Quartz > Kaolinite > illite
	R	Calcite > Quartz > Gismondine > illite
LSYP18	A	Calcite > Quartz-Leucite- Illite
	AC	Calcite > Quartz > Gismondine > Montmorillonite
	C	Calcite > Leucite > Saponite > Montmorionite
	R	Calcite > Quartz > Pillipsite –Enstatite
LSYP19	Ap	Kaolinite > Leucite > Gismondine > Montmorillonit
	C	Calcite > Quartz > Kaolinite > Montmorillonit
LSYP20	A	Calcite > Quartz > Leucite > Enstatite
	CR	Calcite > Enstatite > illite > Montmorillonit
	R	Calcite > Quartz > Leucite > Kaolinite- Saponite
LSYP21	A	Calcite > Quartz > Saponite > Montmorillonite
	AR	Calcite > Quartz > Saponite > Illite > Montmorillonite
	R	Calcite > Saponite > Phillipsite > Montmorillonite
LSYP22	A	Calcite > Quartz > Gismondine > Montmorillonite > Enstatite
	C1	Calcite > perlialite > Illite-Enstatite > Forsterite
	C2	Calcite > Quartz > Gismondine > Montmorillonite > Enstatite
LSYP23	A	Calcite > Quartz > Illite > Gismondine
	C	Calcite > Saponite > Illite > Enstatite
	CR	Calcite > Quartz > Illite > Montmorillonite > Kaolinite
	R	Calcite > Kaolinite > Gismondine > Montmorillonite
LSYP24	Ap	quartz > Gismondine > Enstatite > Montmorillonite
	C1	Calcite > Quartz > Philipsite > Illite
	C2	Calcite > Quartz > Enstatite > Illite
LSYP25	AP	Calcite > Quartz > Illite > Kaolinite
	C	Calcite > Quartz > Kaolinite > montmorillonite
LSYP26	Ap	Calcite > Kaolinite > Philipcite > Montmorillonite
	C1	Calcite > Quartz > Leucite > Montmorillonite
	C2	Calcite > Quartze > Leucite > Augite
	C3	Calcite > Leucite > Enstatite > Saponite
LSYP27	A	(Calcite > Enstatite > Saponite > Montmorillonite.
	AC	Calcite > Leucite > Illite > Montmorillonite
	C	Calcite > Saponite > Kaolinite
LSYP28	A	Calcite > Enstatite > Leucite > Diopside
	AC	Calcite > Quartz > Diopside > Gismondine
	R	Calcite > Leucite > Saponite > Montmorillonite
LSYP29	A	Calcite > Quartz > Gismondine > Montmorillonite
	CR	Calcite > Enstatite > Perlialite > Montmorillonite
	R	Calcite > quartz > Enstatite > Philipsite
LSYP30	A	Calcite > Saponite > Leucite > Illite
	C	Calcite > Saponite > Leucite > Montmorillonite

**Table 6:** Chemical composition of the selected representative soil profiles in the Lattakia region, Syria.

Profile	Horizon	CaO	MgO	Al_2_O_3_	K_2_O	SiO_2_	SO_3_	Na_2_O	Fe_2_O_3_%		
									Total	Non silicate	Silicate
		%									
LSYP01	A	41.78	6.44	14.21	0.52	25.01	0.09	0.18	6.05	0.30	5.75
	B	46.03	6.73	12.21	0.41	21.18	0.06	0.22	5.47	0.34	5.13
	C	39.55	6.60	15.03	0.54	27.56	0.08	0.19	6.22	0.31	5.91
LSYP02	A	32.90	1.50	0.85	0.30	8.70	40.80	10.00	0.60	0.23	0.37
	AC	32.80	1.51	0.80	0.20	8.50	42.00	10.30	0.50	0.24	0.26
	C	32.90	1.60	0.85	0.25	8.60	41.20	10.03	0.52	0.23	0.29
LSYP03	A	29.46	4.65	11.17	0.61	35.93	0.00	0.24	8.05	0.67	7.38
	AC	39.22	5.39	8.52	0.26	36.33	0.00	0.20	6.81	0.36	6.45
	C	32.77	7.29	12.37	0.58	36.39	0.00	0.24	9.21	0.48	8.73
LSYP04	A	22.47	8.18	12.01	0.30	39.35	0.09	0.39	11.63	0.30	11.33
	C1	23.13	5.63	10.37	0.36	42.13	0.06	0.30	11.41	0.32	11.09
	C2	21.69	5.04	12.18	0.40	40.44	0.08	0.30	10.93	0.32	10.61
LSYP05	A	15.29	15.34	12.12	0.37	47.14	0.00	0.70	7.11	0.46	6.65
	C1	21.03	13.73	11.71	0.32	46.34	0.00	0.67	5.90	0.10	5.80
	C2	21.78	11.42	11.81	0.29	46.25	0.00	0.82	5.54	0.10	5.44
LSYP06	A	7.67	9.57	14.36	0.26	47.02	0.00	2.86	11.51	1.95	9.56
	AC	6.42	9.66	14.21	0.30	47.16	0.00	2.76	11.61	1.91	9.70
	R	7.80	8.70	15.46	1.78	48.25	0.00	0.59	10.63	–	10.63
LSYP07	A	14.53	9.08	17.95	0.08	45.51	0.00	2.48	4.56	1.46	3.10
	AC	15.32	11.62	11.73	0.08	45.63	0.3	2.60	3.39	1.41	1.98
	C	18.88	12.03	15.53	0.08	46.12	0.00	1.30	5.52	–	5.52
LSYP08	Ap	28.00	4.94	16.20	0.49	36.96	0.05	0.27	7.75	0.24	7.51
	AC	31.29	4.20	16.72	0.46	34.62	0.05	0.27	7.52	0.25	7.27
	C1	31.05	4.12	16.49	0.53	33.52	0.05	0.27	7.18	0.22	6.96
	C2	29.80	5.38	15.58	0.41	34.18	0.04	0.30	7.93	0.37	7.56
LSYP09	A1	32.60	6.54	14.01	0.67	32.26	0.00	0.24	8.12	1.38	6.74
	(B)	36.39	5.23	13.64	0.73	30.57	0.05	0.23	7.11	1.37	5.74
	BC	34.37	5.45	13.88	0.78	30.57	0.04	0.23	7.33	1.39	5.94
	C1	29.89	5.82	15.99	0.84	35.39	0.05	0.21	8.66	1.37	7.29
	C2	32.97	5.24	11.15	0.71	37.19	0.25	0.21	5.18	1.13	4.05
LSYP10	A	44.69	6.12	9.21	0.47	23.65	0.06	0.16	6.87	0.87	6.00
	AC	46.40	5.11	7.38	0.20	33.61	0.07	0.10	5.84	0.67	5.17
	C	55.13	5.56	6.58	0.16	15.85	0.05	0.13	5.79	0.40	5.39
LSYP11	A	37.87	6.83	8.24	0.10	35.61	0.40	0.60	6.81	0.12	6.69
	R	57.39	7.41	7.96	0.09	13.40	0.04	0.11	7.45	0.17	7.28
LSYP12	A	35.41	6.52	12.02	0.66	29.64	0.05	0.23	8.44	1.28	7.16
	AC	35.54	7.01	11.43	0.67	29.07	0.05	0.23	8.29	1.25	7.04
	C	31.23	7.09	13.27	0.75	33.46	0.05	0.23	9.17	1.21	7.96
LSYP13	A	35.41	6.52	12.02	0.40	29.64	0.00	0.20	8.23	1.22	7.01
	AC	22.54	6.10	11.43	0.41	29.07	0.00	0.21	8.29	1.37	6.92
	C	34.23	7.73	13.27	0.34	33.46	0.05	0.20	7.17	1.11	6.06
LSYP14	A	36.24	5.11	11.59	0.41	32.87	0.00	0.20	6.23	0.83	5.40
	C	33.67	6.47	11.27	0.40	30.27	0.00	0.20	15.03	0.86	14.17
LSYP15	A	9.52	1.94	10.12	0.42	66.07	0.00	0.22	6.29	1.97	4.32
	C1ss	3.78	1.03	19.02	0.57	58.98	0.00	0.32	12.68	1.98	10.70
	C2ss	3.53	0.99	18.75	0.60	59.74	0.00	0.58	13.55	1.98	11.57
LSYP16	A	37.29	6.60	9.39	0.38	33.31	0.00	0.21	8.00	0.35	7.65
	Ap	34.24	6.94	10.27	0.43	34.14	0.00	0.21	8.49	0.12	8.37
	AC	38.38	6.72	9.42	0.37	32.49	0.00	0.21	7.92	0.22	7.70
LSYP17	A	41.92	6.91	7.99	0.26	28.48	0.00	0.19	7.28	0.24	7.04
	AC	23.92	6.85	10.38	0.33	47.26	0.00	0.23	8.98	0.24	8.74
	R	49.53	6.29	7.98	0.21	22.39	0.00	0.18	6.48	–	6.48
LSYP18	A	13.60	2.77	14.27	0.44	55.97	0.00	0.26	16.3	1.38	14.92
	AC	26.99	4.90	11.36	0.31	44.16	0.00	0.21	8.71	1.12	7.59
	C	38.05	7.10	12.32	0.57	28.00	0.00	0.22	6.82	1.38	5.44
	R	47.16	6.27	7.75	0.25	25.20	0.00	0.19	4.85	–	4.85
LSYP19	Ap	28.67	4.73	11.10	0.52	44.83	0.00	0.32	5.15	0.12	5.03
	C	29.60	4.95	10.98	0.37	44.54	0.00	0.33	5.14	0.08	5.06
LSYP20	A	37.10	7.18	8.99	0.28	34.14	0.19	0.17	7.56	0.14	7.42
	CR	39.60	6.90	8.49	0.21	33.00	0.19	0.17	7.02	0.18	6.84
	R	38.07	7.25	9.43	0.00	32.82	0.12	0.17	7.61	0.12	7.49
LSYP21	A	43.94	1.59	9.79	0.51	38.98	0.00	0.33	1.90	0.35	1.55
	AR	40.28	6.25	4.50	0.31	29.83	0.09	0.17	7.86	0.17	7.69
	R	51.65	5.85	7.12	0.10	19.97	0.13	0.07	6.16	–	6.16
LSYP22	A	28.02	3.17	12.16	0.68	43.56	0.00	0.30	6.14	1.46	4.68
	C1	25.45	3.16	12.39	0.58	45.44	0.00	0.33	6.22	0.83	5.39
	C2	23.67	3.31	12.63	0.64	45.16	0.00	0.32	6.56	0.88	5.68
LSYP23	A	34.17	3.11	11.67	0.79	41.97	0.00	0.31	5.58	1.48	4.10
	C	42.38	2.79	10.14	0.55	37.06	0.00	0.28	4.13	1.41	2.72
	CR	49.48	0.58	2.23	0.14	37.50	0.13	0.07	1.24	0.24	1.00
	R	50.14	0.76	1.63	0.10	36.91	0.13	0.07	1.18	0.23	0.95
LSYP24	Ap	39.81	2.43	9.20	0.48	40.38	0.00	0.29	3.61	0.21	3.40
	C1	47.23	1.48	7.73	0.41	37.63	0.00	0.30	2.28	0.24	2.04
	C2	17.73	4.06	12.50	0.39	47.87	0.00	0.37	7.02	0.24	6.78
LSYP25	AP	13.01	6.05	13.21	0.40	52.01	0.00	0.41	7.81	0.11	7.70
	C	13.47	6.08	13.38	0.33	51.55	0.00	0.39	7.92	0.16	7.76
LSYP26	Ap	24.69	4.72	11.61	0.40	46.15	0.00	0.36	5.79	0.18	5.61
	C1	39.59	2.85	8.93	0.39	41.83	0.00	0.32	3.24	0.12	3.12
	C2	31.45	4.80	11.01	0.55	43.97	0.00	0.36	5.04	0.20	4.84
	C3	35.35	4.18	10.38	0.43	43.29	0.00	0.33	4.68	0.12	4.56
LSYP27	A	39.20	6.83	9.17	0.38	29.22	0.00	0.24	7.95	0.24	7.71
	AC	49.18	5.91	7.99	0.30	20.31	0.00	0.21	6.79	0.13	6.66
	C	36.17	7.35	10.06	0.41	32.94	0.00	0.24	8.55	0.11	8.44
LSYP28	A	43.89	7.08	9.31	0.30	28.86	0.00	0.23	8.12	0.15	7.97
	AC	41.62	7.62	10.07	0.39	28.47	0.00	0.21	8.81	0.16	8.65
	R	51.63	6.88	8.65	0.28	20.7	0.00	0.22	7.54	–	7.54
LSYP29	A	42.59	7.33	10.94	0.37	28.31	0.00	0.23	8.28	0.73	7.55
	CR	38.51	7.21	11.1	0.38	30.38	0.00	0.23	9.90	1.82	8.08
	R	46.24	6.91	9.48	0.29	27.78	0.00	0.22	7.72	0.84	6.88
LSYP30	A	31.79	7.96	11.73	0.35	35.78	0.00	0.23	8.98	0.17	8.81
	C	32.65	7.77	11.53	0.37	35.51	0.00	0.25	8.77	0.11	8.66

## 2 Experimental design, materials, and methods

A total of 30 representative soil profiles and 60 auger check points were chosen to represent the different physiography and land cover in the costal region of Lattakia, Syria. The auger points were excavated to 90 cm, while the soil profiles were excavated until the rock parent materials, were encountered. Both were fully described in the field following the guidelines given in Ref. [1]. A total of 106 soil and rock samples were collected from different soil horizons and analysed for physicochemical and mineralogical properties in the laboratory. Soil water content was determined using the loss on ignition method (LOI) according to Ref. [2]. Soil samples were fractionated for sand, silt, and clay using the hydrometer method [3] and the percentage of the fractions were used to obtained the soil texture type based on the USDA particle size classification [4]. Soil bulk and particle densities were determined using the core and pycnometer methods, respectively [5]. Soil pH was measured in a 1:2.5 (soil:water) ratio using a digital pH meter (GH Zeal Ltd., Mi150, UK) as described in Ref. [4]. The soil electrical conductivity (EC) was measured in paste extraction using a digital EC meter (GH Zeal Ltd., Mi170, UK) according to Ref. [6]. Gypsum content was determined using the sedimentation method with acetone according to Ref. [7]. Exchangeable Ca^+2^ and Mg^+2^ were determined using the titration method, while exchangeable Na^+^ and K^+^ were measured using a flame photometer (Microprocessor 1382), all extracted with 1M NH_4_OAc (pH = 7.0) according to Refs. [8,9]. Total calcium carbonate equivalent (CCE) was determined using the calcimeter method [7]. Total organic carbon (TOC) was determined using the wet digestion method [10]. Cation exchange capacity (CEC) was determined following the 1M NH_4_OAc (pH = 7.0) extraction method [11]. Free iron oxides were determined using a dithionite-citrate method buffered with sodium bicarbonate [12]. Major and trace element composition in the soil and rock samples were determined using an X-ray fluorescence spectrometer at the General Company for Cement Manufacture and Building Materials (GCCMBM), Tartous, Syria according to Ref. [13]. Mineralogy for selected clay and rock samples was determined using an X-ray diffractometer (MAXima_X XRD-7000, Shimadzu, Japan) link to PC-APD diffraction software at the General Organization for Geology and Mineral Resources (GOGMR), Damascus, Syria. The XRD patterns were interpreted following the guidelines provided by Ref. [14]. Petrographic microscope (Hundwetzlar, H600 LL, Germany) was used to identify the surface micromorphology of the rock samples at the Geology Department, College of Science, Tishreen University, Lattakia, Syria. The soils were classified based on their properties using *Soil Taxonomy* [15].
